# Monthly Summary

**Published:** 1891-06

**Authors:** 


### Monthly Summary.

AMPUTATION OF THE FOREARM UNDER COCAINE.—Dr. E. C. Rhodes, of Sisson, Cal., reports the following interesting and instructive case: Thos. S., a short stout, robust, muscular laborer, of a lymphatic temperament, was brought from Wright's mill to my office in the forenoon of June 30, 1890. His right hand was completely crushed as far as the wrist, the result of being caught between the drawheads of two loaded cars while attempting to couple them. His face was ashen color, and he seemed faint and distressed from the effects of the shock.

His heart and lungs being apparently in good condition, I decided to administer chloroform, and began to give it from a paper cone containing some absorbent lint on which was poured about a drachm of the anæsthetic, the cone being about 1 inch from the face, so as to give him plenty of air. After two or three inhalations respiration stopped, while the pulse remained good. On taking away the cone and striking the thorax several times respiration re-appeared, but upon replacing the cone it again stopped, each process being repeated three times, after which I laid the chloroform aside, and, not having any ether in the office, concluded to try cocaine.

Filling a 20-minim hypodermatic syringe with a 5-per-cent solution, I injected 5 minims into the dorsal and 5 into the palmar surface of the wrist, just opposite the posterior side of the head of the radius. No bandage, compress, or tourniquet was used above. After waiting five minutes I repeated the injections, same amount and very nearly in the same localities, and immediately began the operation without professional assistance. The patient reclined in the operating chair, looking away, and talked all during the operation, not complaining of pain except during division of the deep tissues on the palmar side of the wrist. After completing the amputation, I applied a 10-per cent. solution of cocaine to the open wound for about two minutes before closing and dressing it.

Now comes what seems to me the only part of the case which merits much consideration. The patient arose from the chair, expressed himself as feeling very much better, walked out of the office, got into the buggy, rode 2½ miles to the mill, ate a good big dinner, and never kept his bed a day nor missed a meal from the effects of the operation. The shock had disappeared. Did the cocaine cause the disappearance? I would like to have the opinion of others on the subject.—*Occidental Medical Times*, March, 1891, p. 121.

---

SENSITIVE DENTINE.—Dr. Williams obtunds sensitive dentine by allowing a simple solution of chloride of lime to remain a short time in the cavity. For the general obtunding of sensitive dentine he has the patient rinse the mouth with dilute lime water, not strong enough to be caustic. He claims to have used these before ether was thought of for that purpose, and says he has obtained good results from them.

